# Production, Characterization, and Sensory Profiling of Novel Pepper Distilled Spirit

**DOI:** 10.1155/2021/9979115

**Published:** 2021-09-26

**Authors:** Yap Chin Ann, Foo See Wei, Gilbert Yeo, Teow Wei Ping, Lawrence Tuah

**Affiliations:** ^1^Research and Development Division, Malaysian Pepper Board, Lot 1115 Jalan Utama, Pending Industrial Area, 93916 Kuching, Sarawak, Malaysia; ^2^Winepak Corporation (M) Sdn. Bhd. No.12, Jalan Utama 2/20, Taman Perindustrian Puchong Utama, Seksyen 2, Batu 14, Jalan Puchong, 47100 Selangor, Malaysia

## Abstract

This study described the production method of novel whiskey using black pepper berries as raw material including the determination of chemical composition and sensory profile of this distillate. The production process consisted of production of fermentation medium via hydrothermal treatment, followed by fermentation and distillation. The incorporation of hydrothermal extraction process makes this whiskey production process differ from the existing commercial spirit production protocol. Chemical composition analysis showed that there were 12 main volatile compounds that contribute to the aroma profile of this pepper whiskey which consisted of 1 aldehyde group, 3 ester groups, 2 phenolic compounds, and 6 alcohol groups. All of these compounds are presented at different concentrations that are able to give pleasant and signature aroma to this spirit. A 10-member descriptive analysis panel conducted showed that 7 descriptors can be used to define the organoleptic quality of this whiskey, namely, clarity and brilliance (visual), fruity and flora (aroma), spiciness and sweetness (taste), and fruity mouthfeels. In conclusion, pepper berries can be used as raw material for pepper whiskey production and having organoleptic quality acceptable for human consumption.

## 1. Introduction

Black pepper, one of the major commodities in Malaysia belonging to genus piper and the family Piperaceae, is an economically important spice that has been well known as “the king of spices.” Its berries are popular throughout the world and are rich in both macronutrient and micronutrient [1]. Pharmacological studies have shown that black pepper has long been known as a carminative, a substance that helps prevent intestinal gas, a property that can stimulate the release of HCl for digestion improvement [2]. In addition, it also has demonstrated impressive diaphoretic properties, diuretic properties, and antioxidant properties besides stimulating the breakdown of fat cell for body slimming purposes [3].

Black pepper plays an important role in the economy of Sarawak, Malaysia, and almost 35,000 households are dependent on pepper as their livelihood. The drastic drop of pepper price due to oversupply scenario led to extensive research on product diversification for enhancing more pepper utilization. Thus, the development of novel pepper-based products is deeming necessary such as pepper whiskey and pepper beers.

Whiskey is one of the famous alcoholic beverages obtained directly from fermentation of cereal grain and fruits before being aged in wooden casks. Nowadays, whiskey has become an important income earner for many countries, for instance, Scotland, the USA, Japan, and Canada [4]. The high volatile compound contents as well as high starch content make it suitable for whiskey brewing. However, none of the reports on pepper whiskey was reported. Therefore, this whiskey is still considered scarce in the local and international whiskey industry and markets.

The characteristics of distilled alcoholic beverage are dependent on the presence of volatile compounds that arise during fermentation, distillation, and aging processes. Volatile compounds are the pivotal sensory indicator and quality parameters for alcoholic beverage profiling [5]. Even though these compounds only contributed to only 1% of the total volume, this small fraction gives alcoholic beverages a unique and signature aroma and character [6]. Besides, the sensory of alcoholic beverage attributes also results from raw materials used, type of fermentation yeast, type of aging cast, and maturation duration [7]. The sensory attributes are also one of the important parameters to be evaluated to prevent counterfeiting and/or adulteration and at the same time to give acceptable perspective of the product at the existing market. Therefore, the determination of these compounds is important to ensure the alcoholic beverage is produced consistently with intense odour and flavour. To our knowledge, no report is found in the literature, dealing with pepper whiskeys that are related to the production and chemical characterizations of volatile compound characteristics. Therefore, the aim of this work is to determine the volatile compound composition that contributes to the aroma profile of the pepper whiskey and to determine the aroma descriptor contributing to the sensory quality of this novel alcoholic beverage.

## 2. Material and Method

### 2.1. Raw Materials

Ground black pepper was supplied by the Malaysian Pepper Board (Kuching, Malaysia). Prior to analysis, the samples were air-dried at 60°C to achieve moisture content of 12% before being stored for subsequent analysis. Chemical compositions of ground black pepper for 100 g dry weight are shown in Table 1.

### 2.2. Fermentation Yeast and Inoculation Preparation

*Saccharomyces cerevisiae* (ELB-181), supplied by Winepak Corporation (M) Sdn. Bhd, was used in this study. This yeast was selected due to its satisfactory fermentative capacity, rapid growth, and easy adaptation. The yeast was maintained at 4°C on yeast extract peptone dextrose medium (YEPD) whose composition consisted of 5 g/L yeast extract, 50 g/L glucose, and 10 g/L peptone. The mixtures were allowed to grow under stirring condition at 200 rpm at 30°C for 24 hours.

For inoculation preparation, the yeast culture was transferred to Erlenmeyer flasks containing 30 g/L of glucose, 3.0 g/L of (NH)_4_HPO_4_, 1.0 g/L of MgSO_4_·7H_2_O, and 3.0 g of yeast extract. The mixtures were then allowed to grow under aseptic condition at 30°C for another 24 hours. The remaining solids after centrifugation were collected and suspended with a fermentation medium for the fermentation process to take part.

### 2.3. Preparation of Fermentation Medium and Fermentation Process

The preparation of a solid fermentation medium of ground black pepper was carried out using a hydrothermal process, in accordance to the method described by Sampaio et al. [8] with some modification. The ground black pepper and malted barley were cleaned and soaked with 2-time volume of 80°C boiled water for approximately 2 hours. After post soaking, approximately 10 g of *α*-amylase was added to the mixture for saccharification at pH 5.5, 35°C for 45 minutes. Subsequently, double gauze was applied for saccharified mixture separation before the mixture is being transferred into a 10 L bioreactor (Biostat C®; B. Braun Biotech International, Melsungen, Germany). Once the prepared sugar was subjected to sterilization at 121°C for 15 minutes, the mixtures finally undergo fermentation processes by inoculation with yeast suspension prepared earlier. The entire fermentation system was maintained at 30°C and performed under anaerobic conditions for approximately 5 days. Samples were aseptically withdrawn from the fermenter to determine the fermentation end via a hydrometer.

The biomass-free fermented broth subsequently undergoes a double distillation process in 10 L Charentais potstill. During distillation, approximately 20 mL of distilled sample was collected at 12-hour interval and the alcohol content was determined. The samples were then blended to obtain 3 fractions of spirit with different ethanol contents: fraction 1: >70 mL/100; fraction 2: 70-40 mL/100 mL; and fraction 3: <40 mL/100 mL). Fraction 2 with ethanol content of 40 mL/100 was collected and matured in 5 L oak casks for three years. An aged whiskey was finally added with 50% of ultrapure water and was stored in glass bottle with caps for subsequent analysis.

### 2.4. Analytical Methods

During the fermentation process, the fermented broths were randomly collected at 12-hour interval, followed by 10 minutes of centrifugation at 5000g to remove cellular debris. The total reducing sugar content presences in the fermented broth were determined using the 3,5-dinitro-salicylic acid method [9]. The ethanol concentration was determined by using high-performance liquid chromatography (HPLC) on Thermo Scientific™ Ultimate 3000 (USA) model chromatograph instrument equipped with a refractive index detector (Thermo Scientific™) with some modification [10].

The major volatile compounds were analysed using HS-SPME-GCMS via the Q Exactive GC system [11]. Sample introduction was performed using a Thermo Scientific™ TriPlus™ RSh Autosampler, and chromatographic separation was obtained with Thermo Scientific™ TRACE™ 1310 GC and a Thermo Scientific™ TraceGOLD™ TG-5SilMS 30 m × 0.25 mm I.D.×0.25 *μ*m filled capillary column with a 10 m integrated guard. Additional details of instrument parameters are shown in Tables 2 and 3. The unknown compounds were subsequently compared with mass spectral data of Wiley Registry-76715-0002, 10^th^ Ed (Thermo Scientific™, USA). The quantification of volatile compounds was performed by comparing their chromatogram peak intensity with that of the 4-nonanal equivalent (internal standard). All the analytical determinations were carried out in triplicate.

### 2.5. Sensory Analysis

A panel of 10 experienced assessors evaluated the sensory characteristics of visual, taste, aroma, and after-taste of the final pepper whiskey, using a vocabulary of 24 terms on an intensity scale of 0-9, where 0 indicated that the descriptor was not perceived and 9 indicated the sample has very high intensity. The samples were served in Glencairn glass (ISO testing glass) covered with watch glass and assess in individual booths under white lighting conditions. During the analysis, each sensory assessor was given approximately 30 mL of sample for visual, smell, and taste. The descriptors were determined by comparing the geometric mean (GM) of each descriptor. GM^2^ was calculated as *F* × *I*, where *F* = number of time descriptor being mentioned/total number of time descriptor being mentioned (expressed as percentage) and *I* = sum of intensities given by the whole panel for a descriptor divided by the maximum number of intensity (expressed as percentage).

To determine the relationship among sensory panelists that contributes to the aroma content of the whiskey, the principal component analysis was applied. This method served as a tool for best descriptor discrimination among panelists.

## 3. Results and Discussion

### 3.1. Fermentation and Distillation

Prior to fermentation, the ground black peppers were submitted for hydrothermal reaction aiming to produce a fermented medium with a pleasant pepper aroma and spiciness. These aromas are considered as an important signature character to differentiate the existing commercial whiskey with this novel pepper whiskey. These distinguishing features become economically important to ensure no counterfeiting or adulteration takes place. Even though this method produced a remarkable signature aroma, the total reducing sugar generated was too little for subsequent analysis. This method only produces approximately 12.3 g/L of sugar which is 15-20-fold lower than the standard fermentation medium [12]. The low reducing sugar detected in the fermentation medium might probably be due to low degradation of starch. In general, starch in barley and black pepper is like most cereals and is the most abundant component within the grain. To achieve complete fermentation performances in brewing, the conversion of starch to reducing sugar must be at maximum with sugar content of >50 g/L [12].

In order to increase the concentration of reducing sugar in the fermentation medium, additional effort was made to modify the hydrolysis method of raw material for maximizing the sugar extraction. An extra step for increasing the degradation activity of starch was conducted via the addition of amylase enzyme. Through this step, the concentration of reducing sugar was increased to almost 100.6 g/L even though it would increase the overall spirit production cost.

**Figure 1:**
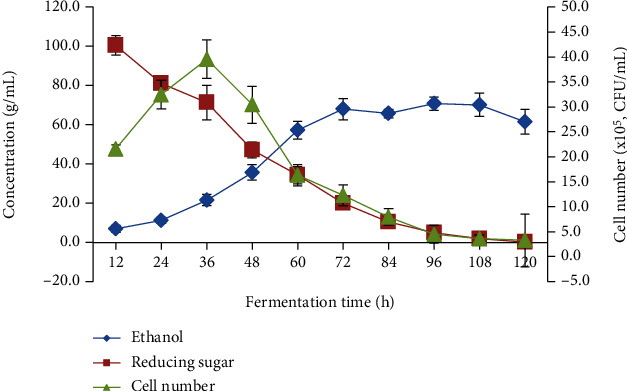
10 L fermentation profile of ethanol production, reducing sugar consumption and cell growth of yeast from ground black pepper fermented medium. Data correspond to mean (±SD) for three independent experiments.

The kinetic behaviour of reducing sugar consumption, ethanol production, and total cell growth is shown in Figure 1. After 12 hours of lag phase, the concentration of ethanol production was increased rapidly and reached the maximum concentration of 67.9 g/L at 72 hours after fermentation with a corresponding yield of 0.94 g/L/h. Similar finding was also being recorded by a research report by Sampaio and Gonçalves [13] who stated the fermentation of spent coffee ground within 10 L fermentation enables to produce ethanol concentration ranged between 0.7 and 0.9 g/L/h. Besides, the concentration of ethanol produced is also at a par with other sources of raw material, e.g., *Batris gasipaes* Kunth and *Spondias mombin* with an ethanol yield of 0.89 and 0.96 g/L/h, respectively [14, 15]. This finding indicated that the hydrothermal process implemented in this project would be suitable for large-scale production of pepper whiskey.

### 3.2. Volatile Compounds

**Figure 2:**
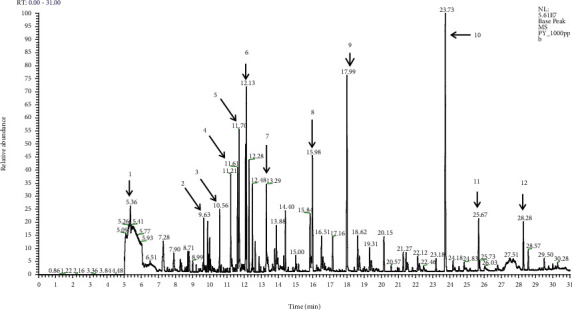
Chromatogram of volatile compound extraction from pepper whisky: 1: acetaldehyde; 2: ethyl acetate; 3: hexyl acetate; 4: methanol, 5: n-propanol, 6: iso-butanol; 7: ethyl butanoate; 8: n-butanol; 9: 2-methyl-1-butanol; 10: 3-methyl-1-butanol; 11: piperine; 12: caryophyllene.

The total ion chromatograms of pepper whiskey are shown in Figure 2, and the major compounds and their respective concentration are summarized in Table 4. A total of 69 volatile compounds were detected and quantified. Among 69 compounds, 31 compounds identified were esters, 15 compounds were alcohols, 10 compounds were acids, 6 were carbonyls, 3 compounds were aldehydes, and the remaining 4 were phenolic compounds. Most of these compounds are important parameters for an alcoholic beverage and must be present in sufficient quantity to provide unique character to the spirit. A similar report has been previously reported in Scotch whisky, Japanese whiskey, and distilled liquor [16]. However, the total concentrations of each volatile compound were significantly different due to variations in production protocol, raw materials use, maturation duration, and aging casks [17–19].

Among the major volatile compounds identified in pepper whiskey, alcohols were the most abundant volatile components that attributed to the signature aroma and taste of pepper whiskey which contributed to almost 82.29% of total major compounds detected. The high concentration of methanol, n-propanol, iso-butanol, n-butanol, 2 methyl-1-butanol, and 3 methyl-1-butanol contributed the greatest proportion of pleasant fruity, sweet, and floral aroma of the pepper whisky. These results were similar to results reported by Swiegers et al. [20] who stated that the volatile alcohol is the major parameter affecting the aroma properties of fermented spirits. The high alcoholic content of spirit is also being reported in grape wines [21], Scotch whisky [22], and Japanese whiskey [23]. By referring to the literature, alcoholic beverages with concentration greater than 1 unit are considered as quality spirit drink [24]. By considering this aspect, it can be concluded that this pepper whiskey is a quality spirit with equivalent quality with existing marketed whiskey and having quality acceptable for human consumption.

Besides alcohol, ethyl acetate, hexyl acetate, and ethyl butanoate (esters) were other major volatile compounds that are present in the high concentration which contribute to almost 11.45% of main compounds detected. These compounds were generally contributed to the fruity and floral aroma of the spirit [25]. In general, the concentration of ester content is considered low as compared to alcoholic compounds and this finding is expected to cause too high concentration of esters in the whiskey leading to poor flavour quality. In accordance with Apostolopoulou et al. [26], spirit with ester concentration above 150 mg/L provides a feature of deterioration to the beverage.

Other major volatile compounds, including acetaldehyde, were also present in pepper whiskey with relatively low concentration (17.66 mg/L). This compound has been reported to provide a pungent and ethereal aroma to pepper whiskey. The concentration of acetaldehyde in various alcoholic beverages has recently been determined to be within the ranges of 0–1000 mg/L in spirits [27–29]. According to Liu et al. [30], higher acetaldehydes in spirit adversely contribute to the unpleasant aroma of spirit when present in concentration higher than 1000 mg/L. By considering both of these factors, both esters and acetaldehyde are within appropriate concentration that may be considered as primary flavour development material for this spirit.

A low content (lower than 5%) of piperine and caryophyllene, phenolic compounds were observed in pepper whiskey with concentration of 5.62 mg/L and 3.12 mg/L, respectively. These compounds are only available in this pepper whisky and contribute to the spiciness and pungent aroma of samples. This finding is expected as black pepper berries were the main raw material for pepper whiskey production. Among the identified volatile compounds from black pepper berries, the main components were found to be caryophyllene (18.4%) and piperine (13.0%, 14.0%).

Figure 2 also summarizes the minor volatile compounds that are present in the pepper whiskey. Although the concentration of these compounds is considered low, the role as whisky flavour developer is undeniable. In fact, this approximately 1% of minor volatile compounds is necessary for the development of new aroma even though they are presented in small quantity. These small amounts of aroma character enable the whiskey producer to distinguish one type of alcoholic distillate from another [23]. The most abundant minor volatile compounds that were present in pepper whiskey were acids. A range of these acids was isolated from pepper whiskey including octanoic acid, dodecanoic acid, hexanoic acid, ellagic acid, and vanillic acid. These acids were likely transferred from freshly made oak barrels to pepper whiskey. Similar compounds were also being detected on Bourbon, Armagnac, Rum, etc. [31]. These compounds are responsible for enhancing the antioxidant capacity of whiskey tested, suggesting that moderate alcohol consumption may improve human health [32]. However, it is worth remembering any possible health benefit is going to be redundant if consumed in large quantities.

### 3.3. Sensory Evaluation

Sensory characteristics are the most important parameter to evaluate the quality of spirit produced, among which sensory appraisal is a commonly accepted method. In this study, a 10-member descriptive analysis panel was performed for the establishment of spirit pleasant descriptors. A significant difference was observed in different quality attributes, such as visual, aroma, taste, and mouthfeels.  Table 5 shows the sensory descriptors of pepper whiskey based on the percentage of geometric means (GM).

A total of 15 sensory descriptors were used in this experiment for pepper whiskey sensory profiling. Two were visual analysis, 6 were aroma profile, 4 were taste profile, and 3 were mouthfeels. Under visual analysis, it was found out that both clarity and brilliance descriptors demonstrated maximum frequency level (100%) with intensity *I* (%) above 80%. This finding indicated that the majority of panelists accept the physical appearance of this whisky.

The fruity was the strongest in the aromatic series with the geometric mean value of 92.74% followed by the flora descriptor (73.48%). The remaining aroma descriptors were considered low with GM values < 50% (woody (44.28%), peaty (26.72%), feinty (8.49%), and sulphury descriptor (4.89%)). The high fruity and floral aromas are mainly due to the present high alcoholic and ester volatile compounds such as ethyl acetate, hexyl acetate, methanol, and n-propanol. This finding was consistent with previous results of Stupak et al. [33] who stated that the alcohol and ester are the main elements that contribute to the fruity flavour of Chinese chestnut whiskey.

In general, the majority of panelists expressed their concern on the spiciness of the whiskey sample with the GM value of 95.92%. This was in agreement with the finding of previous research that alcohol-containing pepper berries tend to be spicier due to the presence of piperine and caryophyllene compounds. Another important descriptors that contribute to the taste profile of pepper whiskey are sweetness. The relatively high GM percentage of sweetness descriptor (76.84%) indicated that this whiskey does contain high sugar content suggesting that reduction in sugar content is necessary for better acceptance among consumers. However, the contribution of smokiness descriptor was relatively low with a GM value of 8.49%. Accordingly, this aroma characteristic was not very obvious.

In terms of mouthfeel, fruity was the main descriptor to describe this spirit with the GM value of 91.10%. This finding was tally with aroma profile with a fruity descriptor as the main aroma contributor to this pepper whiskey. The remaining descriptors such as astringent and bitterness were not obvious. In accordance to International Organization for Standardization (ISO Norm 11035, 1994), only the descriptors greater than 50% were considered as main parameters used to describe the spirit. By looking at this aspect, the pepper whiskey sensory characters can be defined by these 7 descriptors (>50%), including clarity and brilliance (visual), fruity and floral (aroma), spiciness and sweetness (taste), and fruity (mouthfeel).

### 3.4. PCA of Sensory Attributes Pepper Whisky

**Figure 3:**
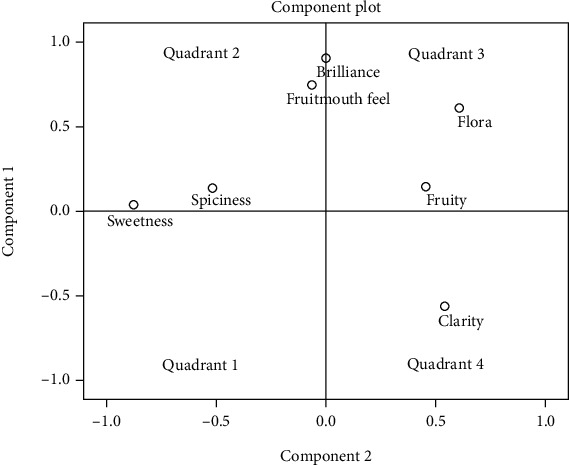
Principle component analysis of preference descriptors (GM > 50%) by a panelist.

SPSS version 16.0 software was used in this study for evaluating the spirit descriptors by each one panelist by using principal component analysis (PCA). For this analysis, only descriptors with GM > 50% were selected for PCA evaluation. It was found out that the maximum variance obtained was at 94%. Through this PCA score, it can be concluded that this pepper whiskey is within the acceptable range among panelist and is suitable for consumer consumption. Two-dimensional analysis of principal component analysis showed that the PC 1 and PC 2 were accounted for 54.54% of the variance and 12.37% of the variance, respectively, in 7 variable systems. The two-dimensional component plots have been generated only for PC 1 and PC 2 as shown in Figure 3. In conclusion, the clarity, a visual descriptor was mainly located in the fourth quadrant, while all the aroma profile, fruity and flora descriptors, were presented in the third quadrant (Figure 3), indicating that fruity, flora, and clarity descriptors are characterized as having high values among panelists with high consumption level. Moreover, the model interpretation suggests that this whiskey also demonstrated some negative descriptors like sweetness and spiciness. This phenomenon can be seen in Figure 3 where both of these descriptors are located at the first and second quadrant of the graph. This finding was similar with research reported by Zhao et al. [34] who stated that both fruity and flora were the main aroma descriptors commonly found on commercial whiskeys like Scotch whisky, Japanese whiskey, and Bourbon.

## 4. Conclusion

Results obtained showed that black pepper berries can be used as raw material for alcoholic beverage production. The production process consisted of 3 main steps, namely, hydrothermal extraction, fermentation, and distillation. The hydrothermal extractions were the novel step designs for the preparation of fermentation medium with pepper aroma and flavour. Twelve signature volatile compounds with different concentrations were identified and quantified by using GCMS analysis. The main volatile compounds present in the pepper whiskey consisted of 1 aldehyde, 2 phenolic compounds, 3 ester groups, and 6 alcohol groups. 15-member descriptive analysis panel conducted showed that 7 descriptors can be used to define the organoleptic quality of this pepper whisky, namely, clarity and brilliance (visual), fruity and flora (aroma), spiciness and sweetness (taste), and fruity mouthfeels. In conclusion, pepper berries can be used as important raw material for pepper whiskey production with signature aroma and flavour. Besides, this raw material can also be used for other value-added products, which would give additional value to pepper berries in the biorefinery industry.

## Figures and Tables

**Table 1:** Chemical composition of ground black pepper.

Chemical properties	Composition (g)
Total starch	50.5 g
Dietary fiber	20.3 g
Total protein	16.1 g
Total phenol compounds	1.2 g
Total flavonoids	0.7 g
Piperine	2.1 g
Caryophyllene	1.7 g
*α*-Pinene	0.6 g
*δ*-3-Carene	0.4 g
DL-limonene	0.3 g
*α*-Copaene	0.2 g

Serving size is 100 g.

**Table 2:** GC-MS/MS and injector conditions.

Trace 1310 GC parameter	
Injection volume (*μ*L)	1 splitless
Liner	Single gooseneck
Inlet	250
Carrier gas, (mL/min)	He, 1.2
Oven temperature program	
Temperature 1 (°C)	45
Hold time (min)	1
Temperature 2 (°C)	330
Rate (°C/min)	10
Hold time (min)	5

**Table 3:** Mass spectrometer conditions.

Q executive GC mass spectrometer parameter	
Transfer line (°C)	280
Ionization type	Electron ionization (EI)
Ion source (°C)	230
Electron energy (eV)	70
Acquisition mode	Full scan
Mass range (Da)	50-600
Resolving power (FWHM)	60,000 (*m*/*z* 200)
Lockmass, column bleed (*m*/*z*)	207.03235

**Table 4:** Main aroma compounds extracted from pepper whisky.

Peak No.	Compounds	Aroma character	Concentration (mg/L)	Percentage (%)
1	Acetaldehyde	Pungent, ethereal	17.66	4.19
2	Ethyl acetate	Floral	26.73	6.34
3	Hexyl acetate	Fruity, flora	17.98	4.26
4	Methanol	Alcoholic, fruity	4.86	1.15
5	n-Propanol	Alcoholic, sweet	63.67	15.09
6	Iso-butanol	Fermented, yeast	52.93	12.55
7	Ethyl butanoate	Fruity, sweet	3.59	0.85
8	n-Butanol	Medicine, fruity	1.35	0.32
9	2-Methyl-1-butanol	Malt	64.78	15.36
10	3-Methyl-1-butanol	Burnt	159.54	37.82
11	Piperine	Pungent, spiciness	5.62	1.33
12	Caryophyllene	Pungent, spiciness	3.12	0.74

**Table 5:** Sensory characteristics of pepper whiskey.

Descriptors	*I* (%)	*F* (%)	GM (%)
*Visual*			
Clarity	89	100	94.34
Brilliance	84	100	91.65
*Aroma*			
Fruity	86	100	92.74
Floral	54	100	73.48
Woody	37	53	44.28
Peaty	21	34	26.72
Feinty	6	12	8.49
Sulphury	3	8	4.89
*Taste*			
*Taste*			
Spiciness	92	100	95.92
Sweetness	72	82	76.84
Smokiness	6	12	8.49
Bitterness	3	7	4.58
*Mouthfeel/afterfeel*			
Fruity	83	100	91.10
Astringent	19	73	37.24
Bitter	5	14	10.21

## Data Availability

The data are already included in the manuscript. However, we will provide you detailed information or data upon request.
